# Research on regularized mean–variance portfolio selection strategy with modified Roy safety-first principle

**DOI:** 10.1186/s40064-016-2621-7

**Published:** 2016-06-29

**Authors:** Ebenezer Fiifi Emire Atta Mills, Dawen Yan, Bo Yu, Xinyuan Wei

**Affiliations:** School of Mathematical Sciences, Dalian University of Technology, Dalian, China; School of Business Management, Dalian University of Technology, Dalian, China; Bank of Dalian, Dalian, Liaoning China

**Keywords:** Mean-risk portfolio, Safety-first, Stable portfolio, Sparse portfolio

## Abstract

We propose a consolidated risk measure based on variance and the safety-first principle in a mean-risk portfolio optimization framework. The safety-first principle to financial portfolio selection strategy is modified and improved. Our proposed models are subjected to norm regularization to seek near-optimal stable and sparse portfolios. We compare the cumulative wealth of our preferred proposed model to a benchmark, S&P 500 index for the same period. Our proposed portfolio strategies have better out-of-sample performance than the selected alternative portfolio rules in literature and control the downside risk of the portfolio returns.

## Background

The benefits of portfolio optimization are well known to investors and other stakeholders. Strategies in portfolio selection have been well documented and unquestionably explored in finance and other related areas from a theoretical and much more practical perspective. Undoubtedly, a widely used approach is the mean–variance model by Markowitz (1952). He demonstrates how investors can employ risk-return tradeoff for wealth allocation using utility functions. Investors decide where along an efficient frontier a suitable balance between risk and return exist. Markowitz’s work has become the bedrock of portfolio selection analysis, thereby earning the name “Modern Portfolio Theory”. In a related study, Roy (1952) proposed a safety-first principle, which minimizes the shortfall probability in portfolio selection. For any investor whose general interest is downside risk measure of investment, Roy’s (1952) safety-first principle is much more suitable Chiu et al. (2012).

Under the safety-first principle, instead of employing utility functions, which is hard or even impossible to determine, a known amount of the principal is preserved. Thus, an investor predefines a minimum threshold level of return and opts for a portfolio of assets that attains this preservation of principal. Roy’s principle has over the years been studied. For instance, Bawa (1978) used the principle to maximize income, given the probability that a predefined threshold is greater than income. Haque et al. (2007) also applied safety-first through extreme value theory to portfolios of Mexican and U.S equities. Rachev (2001) showed how the safety-first approach can be more efficient than the stable Paretian approach in portfolio theory.

Levy and Sarnat (1972) and Nawrocki (1999) show how safety-first principle and mean–variance approach are akin. Levy and Sarnat (1972) found out that in the distinctive case where target rate of return is equal to risk-free return, both Roy’s safety-first principle and Markowitz’s mean–variance approach lead to the same optimal portfolio selection strategy. However, both models have several restrictions which include but are not limited to the following: Firstly, both Markowitz and Roy employ symmetry about the mean variance as a risk measure. Variance takes into account the situation in which return exceeds mean value and this does not impact risk. Secondly, transaction costs are absent in safety-first principle and mean–variance approach. According to DeMiguel et al. (2014), their loss in the mean–variance approach with the absence of transaction costs was 49.33 %. Borkovec et al. (2010) pointed out that 40 % of financial market participants attribute the fundamental loss in abnormal return to transaction costs. Lastly, both model frameworks are subjected to parameter uncertainty as returns of assets are considered as deterministic parameters being depicted by a single point estimate which leads to estimation risk.

Variance, as a risk measure, penalizes both under-performance and over-performance equally (Markowitz 1968). However, investors are only worried about underperformance. Roman et al. (2007) obtained a better portfolio by employing three indexes i.e. mean–variance-CVaR in comparison to mean–variance and mean-CVaR. The authors demonstrated that the mean-CVaR portfolio policy results in large variance, which leads to a small Sharpe ratio. Again, the CVaR of the portfolio generating from mean–variance model is large. To eliminate these inconsistencies between the strategies of mean–variance and mean-downside risk models, Roman et al. (2007) proposed to merge CVaR and variance in a multi-objective portfolio selection strategy. Inspired by Roman et al. (2007)’s approach of combining a downside risk measure, CVaR, and variance, we consider a portfolio optimization model with multiple risk measures. Recognizing a need to modify and improve Roy’s safety first principle, we consider merging our improved Roy-safety first approach with variance as the portfolio risk measure.

The construction of a portfolio of investments is a significant problem faced by investors and institutions. A decision ought to be made to allocate weights to each investment with the intention of striking an appropriate balance between returns and risk. In reality, setting up a new portfolio or rebalancing an existing one requires costs to be incurred and must be inclusive in any realistic analysis. We incorporate proportional transaction costs (Kellerer et al. 2000; Muthuraman and Kumar 2006) which are induced by liquidity costs, tax and brokerage fees (Dumas and Luciano 1991; Kellerer et al. 2000; Lobo et al. 2007) into our portfolio selection model.

In the mean–variance and Roy safety-first models, stock returns are considered deterministic and taken as a single point estimate which results in estimation risk or overfitting (Bawa et al. 1979; Merton 1980). A small variation in the input parameters of the standard Markowitz mean–variance approach and Roy safety-first principle usually lead to changes in the structure of the resulting portfolios (Brandt 2009). To reduce the undesired impact of estimation risk or overfitting, models in the context of robustness (Goldfarb and Iyengar 2003; Tütüncü and Koenig 2004), stochastic programming (Rockafellar and Uryasev 2000), factor models (Green and Hollifield 1992; Nagai 2003; Schultz and Tiedemann 2003) and shrinkage estimators (Jorion 1986; Ledoit and Wolf 2004) have been explored. Another method analogous to our work is the modification of portfolio weights by adding regularizers or additional constraints to the portfolio strategy (Jagannathan and Ma 2003; DeMiguel et al. 2009; Brodie et al. 2009). Correcting undesired portfolio weights are as a result of large estimation risk of unknown parameters leading to the achievement of their desired forms and characteristics. Jagannathan and Ma (2003) employed a short sale constraint in the minimum variance framework and found out the non-negativity constraint performs well as those computed with shrinkage estimators and factor models. In DeMiguel et al. (2009), authors added a convex norm ball constraint to the portfolio weight and observed that the norm ball constrained portfolios had better out of sample performance than the portfolio strategies of the naive 1/*N* diversification (Jagannathan and Ma 2003). The $\textit{l}_2$-norm constrained portfolio in general attained higher Sharpe Ratio than $\textit{l}_1$-norm constrained portfolios. We study the weight constrained portfolios by specifying the general norm as squared $\textit{l}_2$-norm ball.

The optimal portfolio of classical Roy safety-first principle and Markowitz’s model in the presence or absence of short sale constraint or stability constraints hold a large number of assets and especially small weights assigned to their proportions. However, holding a large number of assets leads to the investor incurring high transaction costs. Due to this factor and other market and economic frictions, investors often hold only a small number of stocks in their portfolio. This is usually known as a sparse portfolio (a portfolio with few non-zero weights). With sparsity, one pre-sets limit of assets (stocks) with non-zero entries of portfolio allocations.

A method of constructing sparse portfolio termed hard threshold strategy was proposed by Britten-Jones (1999) to statistically test each portfolio weight with a null hypothesis that weight is zero. Using F-test and t-test, Britten-Jones (1999) proposed that if the portfolio weights are statistically not different from zero; assigning zero reduces portfolio risk. Sparse portfolio weights can be derived by cardinality constrained portfolio optimization (CCPO) problem (Chang et al. 2000; Maringer and Kellerer 2003; Ruiz-Torrubiano and Suárez 2015). CCPO takes into account all portfolios of given number of assets and chooses an optimal portfolio. With the inclusion of cardinality constraint, the portfolio selection strategy becomes NP-hard (Moral-Escudero et al. 2006), and standard quadratic program solvers can no longer be adopted to tackle the problem. One resorts to several other methods or relaxations in search of near-optimal solutions at a moderate computational cost. Farrell and Reinhart (1997) suggested classification of assets based on geographical aspect, size, sector, etc. and made a selection of *N* assets from each of these classes. In Pai and Michel (2009), the cardinality constraints were handled via clustering algorithm to reduce the size of the portfolio. More recently, Ruiz-Torrubiano and Suárez (2015) used a memetic approach that combines a genetic algorithm (GA) with an extended set encoding and quadratic programming (QP) in a mean–variance framework to deal with the cardinality constraint. An alternative relaxation method for which we employ in this paper is by constraining the $l_1$-norm (Tibshirani 1996) which is also in connection with the upper bound of the estimation risk as shown in Fan et al. (2012). The convexity nature of this type of regularization makes it more tractable. The use of norm penalty helps investors limit transaction costs and exposure to risky stocks.

As Roy safety-first principle minimizes the chances that the portfolio’s return will fall below the minimum acceptable return, introducing it in the mean–variance model helps control the downside risk of the portfolio return. The modification and improvement of Roy safety-first principle and merging it with variance as a consolidated risk measure in a risk-return framework represents the main novelty of this research paper. In achieving our aim, we minimize our modified and improved Roy safety-first principle and impose the lower constraint on the mean return of the portfolio as in Markowitz’s mean–variance model. We also investigate the portfolio strategy with variance and Roy’s safety first principle as a consolidated risk measure in a mean-risk framework. To address the problem of estimation risk, we constrain the portfolio weights with squared $l_2$-norm and proceed to achieve sparsity via $l_1$-norm heuristic. We will explore the impact of transaction costs on portfolio selection strategies.

The paper is organized as follows. The next section presents our proposed portfolio selection strategies. It’s subsections study Roy safety-first principle and its modification, portfolio revision and stable and sparse portfolio. We perform numerical tests and present computational results of our proposed methods in the subsequent section. Concluding remarks are provided in the last section.

## Proposed portfolio selection strategies

Investors allocate proportions of their capital among the assets they invest in. For the purpose of this study, these proportions are allocated to stocks. We denote by *N* the number of risky assets, *R* is the required return level, and $x^0$ is the initial risky assets before rebalancing: $x^0_k$ is the proportion of capital initially allocated to asset $k, k=1,2,3,...,N$. Let $x,x^b$ and $x^s$ be *N* dimensional vectors of controllable variables: $x_k$ is the portfolio invested in risky asset *k* after rebalancing, $x^b_k$ is the purchases (proportion used) of risky asset *k* and $x^s_k$ is the sales (proportion obtained) of risky asset *k*. The transaction costs incurred when buying risky assets is $c^b$ and that of selling risky assets is $c^s$. The financial portfolio is described by a *N*-dimensional vector of random returns *r*. The portfolio total random return is $R_p=f(x,r)=\sum ^N_{k=1}x_k r_k$, portfolio expected return vector $\mu _p=\mathbb {E}_x f(x,r)=\sum ^N_{k=1}\mathbb {E}_x r_k$, $\sigma _p^2=\mathbb {E}_x(R_p-\mathbb {E}_x(R_p))^2$ as variance of portfolio return and *Q* as the variance-covariance matrix of the portfolio return.

### Roy safety-first principle and it’s modification

Most investors’ aim is to maximize returns and minimize risk. The Roy safety-first principle advocates avoiding extreme losses through the minimization of disaster probability. To optimally construct a portfolio strategy, Roy’s safety-first principle defines a threshold or a minimum acceptable return *R*, below which the portfolio wealth is considered to be a disaster. The best portfolio is one that minimizes the chances that the portfolio’s return, $R_p$, will fall below a minimum acceptable return, *R*. In essence, an investor selects his portfolio by solving this optimization problem:

1 $$\begin{aligned} \underset{x \in \mathbb {R}^{N}}{\text {minimize}}&\quad \mathbb {P}(f(x,r) \le R)\nonumber \\ \text {subject to}&\quad e^Tx=1 \end{aligned}$$

where *e* is a vector with ones as entries and $\mathbb {P}$ is a probability measure. Roy employed Bienayme–Tchebycheff's inequality as the investor is likely not to know the actual probability function and obtained an approximation

$$\begin{aligned} \mathbb {P}(R_p \le R)\le \frac{\sigma _p^2}{(\mu _p-R)^2} \end{aligned}$$

Thus, the optimization problem is reformulated as

2 $$\begin{aligned} \underset{x \in \mathbb {R}^{N}}{\text {minimize}}&\quad \frac{\sigma _p^2}{(\mu _p-R)^2} \nonumber \\ \text {subject to}&\quad e^Tx=1 \end{aligned}$$

The modification to the Roy’s approach we adopt is by using a coherent downside risk measure known as Conditional Value-at-Risk or Expected shortfall as it has a set of desirable properties for a risk measure (Platen and Heath 2006) leading to more accurate estimates of probability. For a detailed study on desirable properties of an ideal risk measure in portfolio theory, we refer the reader to Rachev et al. (2008).

A well-known downside risk measure known as Value-at-Risk focuses on the percentiles of loss distributions and measures the predicted maximum loss at a given probability level. Mathematically it is formulated as $\alpha$-quantile $VaR_\alpha (X)= min \{z\mid (F_X(z)\ge \alpha \}$, where *X* is a loss random variable and $\alpha \in (0,1)$ is the given probability level. Values for $\alpha$ often used are 90 %, 95 % and 99 %. Considering Value-at-Risk (VaR) has undesirable properties such as non-subadditive and non-smooth etc., Rockafellar and Uryasev (2000) introduced a coherent downside risk measure termed Conditional Value-at- Risk (CVaR) and for $\alpha \in (0,1)$ represented it as

$$\begin{aligned} CVaR_\alpha (X)= \int _{-\infty }^{+\infty }zdF_X^{\alpha }(z) \end{aligned}$$

where

$$\begin{aligned} \begin{array}{@{} r @{} c @{} l @{} } &F_X^{\alpha }(z)\,&\,= {\left\{ \begin{array}{ll} 0 & \quad \text {when } z<VaR_{\alpha }(X) ,\\ \frac{F_X(z)-\alpha }{1-\alpha }& \quad \text {when } z \ge VaR_\alpha (X). \end{array}\right. } \end{array} \end{aligned}$$

Equivalently, for $x \in X \subseteq \mathbb {R}^N$ and random vector $r\in \mathbb {R}^N$ which represents the actual portfolio return has a continuous density function *p*(*r*)

$$\begin{aligned} CVaR_\alpha (x)= \frac{1}{1-\alpha } \int _{f(x,r) \ge VaR_\alpha (x)}f (x,r)p(r)dr \end{aligned}$$

First, we will determine the semi-deviation of the random return *f*(*x*, *r*) from the $\alpha$-quantile $VaR_\alpha (x)$. With respect to Bienayme–Tchebycheff's inequality, the following estimate is valid for $VaR_{\alpha )}(x)>R$:

3 $$\begin{aligned} \mathbb {P}\{f(x,r) \le R \}& = \mathbb {P}\{-f(x,r)\ge -R\}\nonumber \\ & = \mathbb {P} \{VaR_{\alpha )}(x)-f(x,r) \ge VaR_{\alpha )}(x)-R\}\nonumber \\& = \mathbb {P}\{\mid f(x,r)-VaR_{\alpha )}(x)\mid \ge VaR_{\alpha )}(x)-R \}\nonumber \\ & \le \mathbb {P}\mid \{\min \{f(x,r)-VaR_{\alpha )}(x), 0\}\mid \ge VaR_{\alpha )}(x)-R \}\nonumber \\ & \le \frac{\ \mathbb {E}_x \mid \min \{f(x,r)-VaR_{\alpha }(x), 0\}\mid }{VaR_{\alpha )}(x)-R} \end{aligned}$$

Let us consider an $\alpha$-quantile

$$\begin{aligned} VaR_{\alpha }(x)= \min \{z\mid \mathbb {P}(f(x,r)\le z)\ge \alpha \}\implies \mathbb {P}\{(f(x,r)\ge VaR_{\alpha }(x)\}=1-\alpha \end{aligned}$$

and a measure of risk

$$\begin{aligned} CVaR_{\alpha }(x)=\frac{\mathbb {E}_x \mid \min \{f(x,r)-VaR_{\alpha }(x), 0\}\mid }{\mathbb {P}\{f(x,r) \ge VaR_{\alpha }(x)\}} \end{aligned}$$

which is termed expected shortfall from the $\alpha$-quantile $VaR_\alpha (x)$ value. The estimate (3) can be written as

4 $$\begin{aligned} \begin{aligned} \mathbb {P}\{f(x,r) \le R \}&\le \frac{ \mathbb {P}\{f(x,r) \ge VaR_{\alpha }(x)\}}{VaR_{\alpha }(x)-R}\frac{\mathbb {E}_x \mid \min \{f(x,r)-VaR_{\alpha }(x), 0\}\mid }{\mathbb {P}\{f(x,r) \ge VaR_{\alpha }(x)\}}\\&\le \frac{(1-\alpha )CVaR_{\alpha }(x)}{VaR_{\alpha }(x)-R} \end{aligned} \end{aligned}$$

Therefore (1) can be reformulated by considering the approximation of the right hand side of (4) and obtain the following

5 $$\begin{aligned} \underset{x \in \mathbb {R}^{N}}{\text {minimize}}&\quad \frac{(1-\alpha )CVaR_{\alpha }(x)}{VaR_{\alpha }(x)-R} \nonumber \\ \text {subject to}&\quad VaR_{\alpha }(x)> R\nonumber \\&\quad e^Tx=1 \end{aligned}$$

Telser (1955) considered a portfolio strategy by maximizing portfolio returns under the the constraint of Roy safety-first principle. He solved the optimization problem:

6 $$\begin{aligned} \underset{x \in \mathbb {R}^{N}}{\text {maximize}}&\quad \mu ^Tx \nonumber \\ \text {subject to}&\quad \mathbb {P}(f(x,r) \le R) \ge {1-\epsilon } \end{aligned}$$

Inspired by Telser’s approach, we constrain (5) with the minimum mean return vector $\mu ^Tx \ge L$ from below where *L* is the lower bound of $u^Tx$, where $L>R$. Thus we obtain the optimization problem :

7 $$\begin{aligned} P_0:\quad \underset{x \in \mathbb {R}^{N}}{\text {minimize}}&\quad \frac{(1-\alpha )CVaR_{\alpha }(x)}{VaR_{\alpha }(x)-R}\nonumber \\ \text {subject to}&\quad \mu ^Tx\ge L\nonumber \\&\quad VaR_{\alpha }(x)> R\nonumber \\&\quad e^Tx=1 \end{aligned}$$

In another approach, we consider variance and the modified Roy’s safety first-principle as a consolidated risk measure in a mean-risk framework. To this end, we propose the optimization problem:

8 $$\begin{aligned} P_1:\quad \underset{x \in \mathbb {R}^{N}}{\text {minimize}}&\quad x^TQx+\frac{(1-\alpha )CVaR_{\alpha }(x)}{VaR_{\alpha }(x)-R}\nonumber \\ \text {subject to}&\quad \mu ^Tx\ge L\nonumber \\&\quad VaR_{\alpha }(x)> R\nonumber \\&\quad e^Tx=1 \end{aligned}$$

The rest of the modifications is geared towards investigating realistic constraints such as transaction costs, sparsity, and stability to $P_0$ and $P_1$ on the financial market.

### Portfolio revision

We consider an extension of problems (7) and (8) in which transaction costs are incurred to rebalance or revise the initial portfolio $x^0$, into an efficient portfolio *x*. A portfolio of investments may require rebalancing on periodical basis because of updated risk, and return information is generated over time. We make the following assumptions on the transaction cost function *c*.

#### **Assumption 1**

The transaction cost function satisfies the following:

(i) *c(x)* is a convex function of *x*
(ii) *c(0) = 0*
(iii) *c(x)*$$\ge 0, \quad \forall x$$

To achieve portfolio $x_k$ from the previous or initial portfolio $x_k^0$, we make a payment of transaction costs $c(x-x^0)$. We incorporate proportional transaction costs (Kellerer et al. 2000; Muthuraman and Kumar 2006; Mitchell and Braun 2013) which are induced by liquidity costs, tax, brokerage fees (Dumas and Luciano 1991; Kellerer et al. 2000; Lobo et al. 2007) into our model. Therefore, proportional transaction cost follows this structure:

$$\begin{aligned} c(x-x^0)=\sum _{k=1}^N c_k \left( x_k-x_k^0\right) \end{aligned}$$

where

$$\begin{aligned} \begin{array}{@{} l @{} l @{} l @{} } &c_k (x_k-x^0_k)\;&\;= {\left\{ \begin{array}{ll} c_k^b (x_k-x^0_k) & \quad \text {if } \quad x_k \ge x^0_k ,\\ c_k^s (x_k^0-x_k) & \quad \text {if } \quad \mathrm{otherwise}. \end{array}\right. } \end{array} \end{aligned}$$

where cost of buying is $c_k^b \>0$ and cost of selling is $c_k^s>0$.

The $P_1$ model with proportional transaction costs $(P_{1t})$ is the optimization problem

9 $$\begin{aligned} P_{1t}:\quad \underset{x,x^s,x^b \in \mathbb {R}^{N}}{\text {minimize}}&\quad x^TQx+ \frac{(1-\alpha )CVaR_{\alpha }(x)}{VaR_{\alpha }(x)-R} \end{aligned}$$

10 $$\begin{aligned} \text {subject to}&\quad \mu ^Tx-\sum _{k=1}^N \left( c^bx_k^b+c^s x_k^s\right) \ge L \end{aligned}$$

11 $$\begin{aligned}&\quad \sum _{k=1}^N x_k +c^b \sum _{k=1}^N x_k^b+c^s \sum _{k=1}^N x_k^s=1 \end{aligned}$$

12 $$\begin{aligned}&\quad x_k=x_k^0+x_k^b-x^s_k, \quad \forall k=1,\ldots ,N \end{aligned}$$

13 $$\begin{aligned}&\quad x_k^b \cdot x_k^s=0, \quad \forall k=1,\ldots ,N \end{aligned}$$

14 $$\begin{aligned}&\quad VaR_{\alpha }(x)> R \end{aligned}$$

15 $$\begin{aligned}&\quad x_k^b\ge 0 ,\quad x_k^s \ge 0, \quad \forall k=1,\ldots ,N \end{aligned}$$

The model $P_{1t}$ minimizes the upper bound estimate (5) w.r.t *x* and superimposes the lower constraint $L \le \mu 'x-\sum _{k=1}^N (c^bx_k^b+c^s x_k^s)$ on the average return after the deduction of transaction costs.

Explaining the constraints with respect to transaction costs, the above optimization problem is subjected to a set of linear constraints. Constraint (10) requires the net return of the portfolio after the deduction of transaction costs to be greater or equal to a threshold level *L*. Constraint (11) is the budget constraint: the capital available to cover transaction costs and shares of stocks. Constraint (12) shows that $x_k$ represents the portfolio position to be chosen explicitly through sold shares $x^s_k$ and purchased shares $x^b_k$ that are rebalanced adjustments to the initial position $x^0_k$ of stock *k*. Constraint (13) and constraint (15) are the complementary constraint and non-negative constraint respectively. They prevent any possibility of concurrent purchases and sales (Dybvig 2005). Note that $P_0$ model with proportional cost $(P_{0t})$ is defined similarly but without the variance term in the objective function.

### Stable and sparse portfolio

In a portfolio selection strategy where the dimensionality of the set of candidate assets is high, sparsity is desired. When the number of assets is large, a non-regularized numerical approach will intensify the effects of estimation risk, leading to an unstable and unreliable estimate of the vector *x*. Typically, portfolio managers want to set up portfolios with suitable balance between risk and return by investing in a small number of assets, thereby limiting their transaction, management, and monitoring costs.

To obtain meaningful and sparse (zero components) results, a regularization procedure is usually adopted. A standard approach is to augment the objective function of interest with a $\textit{l}_0$-norm penalty or adding a cardinality constraint $\Vert x \Vert _0 \le N'$ to optimization problems $P_{0t}$ and $P_{1t}$, where $\Vert x \Vert _0$ is the number of the non-zero entries of *x* and $N'$ is the upper bound limitation of assets to be managed in the portfolio. However, with the inclusion of cardinality constraint, the portfolio selection strategy becomes NP-hard (Moral-Escudero et al. 2006). We therefore impose its equivalent $\textit{l}_1$-norm penalty as employed by Brodie et al. (2009) among others. The $\textit{l}_1$-norm is a convex function of *x*, and such convex relaxation makes portfolio selection strategy more tractable.

To this end, we suggest to evaluate the portfolio weights by

16 $$\begin{aligned} S_{1t}:\quad \underset{x,x^s,x^b \in \mathbb {R}^{N}}{\text {minimize}}&x^TQx+\frac{(1-\alpha )CVaR_{\alpha }(x)}{VaR_{\alpha }(x)-R} +\tau _1 \Vert x\Vert _1\nonumber \\ \text {subject to}&\quad \mu 'x-\sum _{k=1}^N \left( c^bx_k^b+c^s x_k^s\right) \ge L\nonumber \\&\quad \sum _{k=1}^N x_k +c^b \sum _{k=1}^N x_k^b+c^s \sum _{k=1}^N x_k^s=1\nonumber \\&\quad x_k=x_k^0+x_k^b-x^s_k, \quad \forall k=1,\ldots ,N\nonumber \\&\quad x_k^b \cdot x_k^s=0, \quad \forall k=1,\ldots ,N\nonumber \\&\quad VaR_{\alpha }(x)> R\nonumber \\&\quad x^b_k\ge 0 ,\quad x^s_k \ge 0,\quad \forall k=1,\ldots ,N \end{aligned}$$

where the $\textit{l}_1$-norm of a vector $x \in \mathbb {R}^N$ is defined by $\Vert x\Vert _1{:}{=}\sum _{k=1}^N \mid x_k \mid$ and $\tau _1$ is an adjustable parameter that controls the sparsity of the portfolios. Similarly, $S_{0t}$ can be formulated without the variance term in the objective function.

Optimization models $S_{0t}$ and $S_{1t}$ have estimation risk or overfitting problem. The $l_1$-norm penalty expedites sparsity of *x* and leads to a subset of assets receiving zero weights. Such sparsity may result in under-diversification and extreme weights of the portfolio. On the contrary, convex norm ball does not produce sparsity but it can efficiently regularize size of portfolio weight vector. Thus, the norm ball constraint used by DeMiguel et al. (2009) can function as a solution to alleviate the problems of under-diversification and extreme weights of the portfolio aside estimation risk. Following DeMiguel et al.’s (2009) work and specifying the general squared $l_2$-norm under no short sale constraint, we propose to formulate the portfolio weights by

17 $$\begin{aligned} RSMt:\quad \underset{x,x^b,x^s \in \mathbb {R}^{N}}{\text {minimize}}&\quad \frac{(1-\alpha )CVaR_{\alpha }(x)}{VaR_{\alpha }(x)-R}+\tau _1 \Vert x\Vert _1+\tau _2 \Vert x \Vert _2^2 \nonumber \\ \text {subject to}&\quad \mu ^Tx-\sum _{k=1}^N \left( c^bx_k^b+c^s x_k^s\right) \ge L\nonumber \\&\quad \sum _{k=1}^N x_k +c^b \sum _{k=1}^N x_k^b+c^s \sum _{k=1}^N x_k^s=1\nonumber \\&\quad x_k=x_k^0+x_k^b-x^s_k, \quad \forall k=1,\ldots ,N\nonumber \\&\quad x_k^b \cdot x_k^s=0, \quad \forall k=1,\ldots ,N\nonumber \\&\quad VaR_{\alpha }(x)> R\nonumber \\&\quad x^b_k\ge 0 ,\quad x^s_k \ge 0,\quad \forall k=1,\ldots ,N \end{aligned}$$

and

18 $$\begin{aligned} RSMVt:\quad \underset{x,x^b,x^s \in \mathbb {R}^{N}}{\text {minimize}}&\quad x^TQx+\frac{(1-\alpha )CVaR_{\alpha }(x)}{VaR_{\alpha }(x)-R}+\tau _1 \Vert x\Vert _1+\tau _2 \Vert x \Vert _2^2 \nonumber \\ \text {subject to}&\quad \mu ^Tx-\sum _{k=1}^N \left( c^bx_k^b+c^s x_k^s\right) \ge L\nonumber \\&\quad \sum _{k=1}^N x_k +c^b \sum _{k=1}^N x_k^b+c^s \sum _{k=1}^N x_k^s=1\nonumber \\&\quad x_k=x_k^0+x_k^b-x^s_k, \quad \forall k=1,\ldots ,N\nonumber \\&\quad x_k^b \cdot x_k^s=0, \quad \forall k=1,\ldots ,N\nonumber \\&\quad VaR_{\alpha }(x)> R\nonumber \\&\quad x^b_k\ge 0 ,\quad x^s_k \ge 0,\quad \forall k=1,\ldots ,N \end{aligned}$$

where $\tau _1$ and $\tau _2$ are tuning parameters controlling sparsity and stability respectively, with $\Vert x \Vert _2^2 =x'x$ as the squared $l_2$-norm of a vector. We estimate tuning parameters $\tau _1$ and $\tau _2$ by a method of cross-validation. We perform cross-validation for various possible values of the parameters and select the parameter value that produces the minimum cross-validation average error. A combination of the $l_2$-norm penalty and $l_1$-norm penalty is referred to as elastic net (Zou and Hastie 2005).

### Necessary and sufficient conditions for optimal problems

In this section we would like to identify the necessary and sufficient conditions for optimality of problems (17) and (18). We investigate the Karush-Kuhn-Tucker (KKT) conditions for these problems under the assumption of normality and study whether the constrained problems have optimal solutions.

#### KKT conditions for optimal problem

The KKT conditions provide necessary conditions for a point to be optimal point for a constrained nonlinear optimal problem. The system

19 $$\begin{aligned}&x_k=x_k^0+x_k^b-x^s_k, \quad \forall k=1,\ldots ,N\nonumber \\&x_k^b \cdot x_k^s=0, \quad \forall k=1,\ldots ,N\nonumber \\&x_k^b\ge 0 ,\quad x_k^s \ge 0 \end{aligned}$$

has a unique solution

$$\begin{aligned} x_k^b &= \left\{ \begin{array}{@{}l@{\quad }l@{}} x_k-x_k^0, & \quad \text {if} \quad x_k-x_k^0 \ge 0\\ 0, & \quad \text {else}\\ \end{array}\right. \\ x_k^s & = \left\{ \begin{array}{@{}l@{\quad }l@{}} x_k^0-x_k, & \quad \text {if} \quad x_k-x_k^0 < 0\\ 0, & \quad \text {else}\\ \end{array}\right. \end{aligned}$$

i.e.

$$\begin{aligned} \begin{aligned} x_k^b=max\{x_k-x_k^0,0\}\\ x_k^s=max\{x_k^0-x_k,0\} \end{aligned} \end{aligned}$$

From the budget constraint $\sum _{k=1}^N x_k +c^b \sum _{k=1}^N x_k^b+c^s \sum _{k=1}^N x_k^s=1$, we get that $c^b \sum _{k=1}^N x_k^b+c^s \sum _{k=1}^N x_k^s=1-\sum _{k=1}^N x_k$. Implementing the first constraint,$\mu ^Tx-\sum _{k=1}^N (c^bx_k^b+c^s x_k^s)\ge L$ we get that $\mu ^Tx-(1-\sum _{k=1}^N x_k)\ge L$. Thus, RSMt can be represented as

20 $$\begin{aligned} \underset{x \in \mathbb {R}^{N}}{\text {minimize}}&\quad f_1(x)=\frac{(1-\alpha )CVaR_{\alpha }(x)}{VaR_{\alpha }(x)-R}+\tau _1 \Vert x\Vert _1+\tau _2 \Vert x \Vert _2^2 \nonumber \\ \text {subject to}&\quad g_1(x)= \mu ^Tx-\left( 1-\sum _{k=1}^N x_k\right) \ge L\nonumber \\&\quad h_1(x)=c^b \sum _{k=1}^N max\left\{ x_k-x_k^0,0\right\} +c^s \sum _{k=1}^N max\left\{ x_k^0-x_k,0\right\} =1-\sum _{k=1}^N x_k\nonumber \\&\quad g_2(x)= VaR_{\alpha }(x)> R \end{aligned}$$

Similarly, RSMVt can be represented with addition of $x^TQx$ to the objective function.

Let *x* be a regular point for the problem RSMt. Then the point *x* is a local minimum of *f* subject to constraints (20) if there exists Lagrange multipliers $\lambda _1, \lambda _2$ and $\lambda _3$ for the Lagrangian function $L=f_1(x)+\lambda _1 g_1(x)+ \lambda _2 h_1(x)+\lambda _3 g_2(x)$ such that the following are true.

1. $$\frac{\partial L}{\partial x}={(1-\alpha )CVaR'_{\alpha }(x)}{VaR_{\alpha }(x)-R}-\frac{(1-\alpha )CVaR_{\alpha }(x) VaR'_{\alpha }(x)}{(VaR_{\alpha }(x)-R)^2}+\tau _1 c^1 +2\tau _2x-\lambda _1(\mu +I^{N \times 1})+ \lambda _2 c^2-\lambda _3 VaR'_{\alpha }(x)=0$$
2. $$\lambda _1(L-\mu ^Tx-(1-\sum _{k=1}^N x_k)=0$$
3. $$\lambda _3(R-VaR_{\alpha }(x))=0$$
4. $$\lambda _1,\lambda _3 \ge 0$$
5. $$\mu ^Tx-(1-\sum _{k=1}^N x_k)\ge L$$
6. $$c^b \sum _{k=1}^N max\{x_k-x_k^0,0\} +c^s \sum _{k=1}^N max\{x_k^0-x_k,0\}=1-\sum _{k=1}^N x_k$$
7. $$VaR_{\alpha }(x)> R$$

where

$$\begin{aligned} c_k^1 &= \left\{ \begin{array}{@{}l@{\quad }l@{}} 1, & \quad \text {if } \quad x_k>0\\ -1, & \quad \text {if } \quad x_k<0\\ \in [-1,1], & \quad \text {if } \quad x_k=0\\ \end{array}\right. \\ c_k^2 & = \left\{ \begin{array}{@{}l@{\quad }l@{}} c^b, & \quad \text {if }\quad x_k>x_k^0\\ -c^s, & \quad \text {if } \quad x_k<x_k^0\\ \in [-c^s,c^b], & \quad \text {if }\quad x_k=x_k^0\\ \end{array}\right. \end{aligned}$$

##### *Remark 1*

Since the function $h_1$ in (20) is linear and the functions $g_1$ and $g_2$ are convex, then the feasible region $\Omega = \{x : h_1,g_1,$ and $g_2\}$ is a convex set. On the other hand, $f_1$ is a convex function subject to the variable *x*. We see that any local minimum for problem (20) is a global minimum too and KKT conditions are also sufficient.

Similarly, the above KKT conditions holds for RSMVt for when $f_1(x)$ in (20) is $f_2(x)=x^TQx+\frac{(1-\alpha )CVaR_{\alpha }(x)}{VaR_{\alpha }(x)-R}+\tau _1 \Vert x\Vert _1+\tau _2 \Vert x \Vert _2^2$ and when the following are true

1. $$\frac{\partial L}{\partial x}=2Qx+{(1-\alpha )CVaR'_{\alpha }(x)}{VaR_{\alpha }(x)-R}-\frac{(1-\alpha )CVaR_{\alpha }(x) VaR'_{\alpha }(x)}{(VaR_{\alpha }(x)-R)^2}+\tau _1 c^1 +2\tau _2x-\lambda _1(\mu +I^{N \times 1})+ \lambda _2 c^2-\lambda _3 VaR'_{\alpha }(x)=0$$
2. $$\lambda _1(L-\mu ^Tx-(1-\sum _{k=1}^N x_k)=0$$
3. $$\lambda _3(R-VaR_{\alpha }(x))=0$$
4. $$\lambda _1,\lambda _3 \ge 0$$
5. $$\mu ^Tx-(1-\sum _{k=1}^N x_k)\ge L$$
6. $$c^b \sum _{k=1}^N max\{x_k-x_k^0,0\} +c^s \sum _{k=1}^N max\{x_k^0-x_k,0\}=1-\sum _{k=1}^N x_k$$
7. $$VaR_{\alpha }(x)> R$$

where

$$\begin{aligned} c_k^1 & = \left\{ \begin{array}{@{}l@{\quad }l@{}} 1, & \quad \text {if } \quad x_k>0\\ -1, & \quad \text {if } \quad x_k<0\\ \in [-1,1], & \quad \text {if } \quad x_k=0\\ \end{array}\right. \\ c_k^2 & = \left\{ \begin{array}{@{}l@{\quad }l@{}} c^b, & \quad \text {if }\quad x_k>x_k^0\\ -c^s, & \quad \text {if }\quad x_k<x_k^0\\ \in [-c^s,c^b], & \quad \text {if } \quad x_k=x_k^0\\ \end{array}\right. \end{aligned}$$

##### *Remark 2*

Since the feasible region $\Omega = \{x : h_1,g_1,$ and $g_2\}$ in (20) and the objective function $f_2(x)$ are convex, then the feasible region is a convex set. We can see that the KKT conditions are also sufficient and any local minimum for problem (20) with objective function $f_2(x)$ is a global minimum as well.

## Empirical application

### Data and models

In this section, we use optimization models RSMt (17) and RSMVt (18) to construct optimal portfolios and evaluate out-of-sample performance using stocks traded on New York Stock Exchange (NYSE). Historical daily returns of 500 randomly selected stocks over the period January 2003 to December 2015 were extracted from Yahoo Finance. The risk-free rate is proxied by the 6-month US Treasury Bill rate. The selection criterion of the random sampling of stocks is based on stocks being traded throughout the evaluation period. We also use S&P 500 index daily stock price data to test the robustness of our results over the same time period even though 21 % of randomly selected stocks are components of S&P 500. We chose a short term period as distribution of stock prices tend to change shape over time. To solve the portfolio strategies, we consider the lower bound of mean return as threshold return level. We compute initial positions $x^0_k,k=1,\ldots ,N$ for constructing portfolios for January 2006 to December 2008 and January 2009 to January 2015 by solving classical Roy safety-first optimization problem:

21 $$\begin{aligned} {\text {minimize}}&\quad \frac{\sigma _{\bar{x}}^2}{(\mu _{\bar{x}}-R)^2} \nonumber \\ \text {subject to}&\quad e^T{\bar{x}}=1\nonumber \\&\quad \bar{x} \in \mathbb {R}^+_N, \end{aligned}$$

and by setting $x_0={\bar{x}}^*$, with ${\bar{x}}^*$ denoting the optimal solution of the above model.

Two portfolio strategies proposed in this research work, RSMt and RSMVt are compared against these existing ones in literature: (1) sample minimum variance without short sale constraint portfolio (minVu) (2) sample minimum variance short sale constrained portfolio (minVc) (3) sample mean–variance approach (MV) (4) naive equally-weighted (1/*N*) portfolio (5) $l_1$-penalized mean–variance model ($l_1$MV) (Brodie et al. 2009) (6) linear combination of sample tangency portfolio, sample minimum variance portfolio and 1/*N* portfolio (TMN) (Tu and Zhou 2011) (7) minimum variance portfolio resulting from using a diagonal covariance matrix (VD) (Kirby and Ostdiek 2012), refer to Table 1.

**Table 1:** List of proposed and selected alternative portfolio strategies

Symbol	Description
RSMt	$l_1$- Squared $l_2$ penalized mean-Roy safety-first portfolio
RSMVt	$l_1$- Squared $l_2$ penalized mean–variance-Roy safety-first portfolio
minVu	Sample minimum variance without short sale constraint portfolio
minVc	Sample minimum variance with short sale constraint portfolio
MV	Sample mean–variance portfolio
1/*N*	Equally-weighted portfolio
$l_1$-MV	$l_1$ penalized mean–variance portfolio with short sale constraint (Brodie et al. 2009)
TMN	Linear combination of sample tangency portfolio, sample minimum variance portfolio and 1/*N* portfolio (Tu and Zhou 2011)
VD	Minimum variance portfolio resulting from using a diagonal covariance matrix (Kirby and Ostdiek 2012)

We employ a 6-month rolling estimation window for parameter estimations and construct portfolios on a sub-sample periodical basis. To obtain the portfolio for January 2006 to December 2008, we use January 2003 to December 2005 data to construct the initial positions via the classical Roy safety-first optimization problem. For January 2009 to December 2011, we set the portfolio weights from January 2006 to December 2008 as initial positions. We then set the portfolio weights from January 2009 to December 2011 as initial positions to construct portfolios for January 2012 to December 2015. To reflect the true risk of the portfolio, we use January 2006 to December 2008 data as initial positions via the classical Roy safety-first optimization problem to construct portfolios for January 2009 to December 2015. Portfolio revision is made on a monthly basis since in reality they are related to low transaction, management and monitoring costs.

### Evaluation criteria

With regards to the return that a portfolio can achieve, we calculate and present the annualized out-of-sample Sharpe ratio with or without transaction cost. Sharpe ratio is defined as the ratio of the expected excess return to standard deviation of portfolio return (Sharpe 1966). The expected excess return is the difference between the return of the portfolio and the return obtained through a risk-free security. Mathematically, we can define Sharpe ratio as $SRatio = \frac{\rho -\rho _f e}{\sigma }$, where $\rho _f$ is the return from the risk-free security (6-month US Treasury Bill rate) and $\sigma$ is the standard deviation of portfolio return. Regarding portfolio risk, we consider the risk reduction which is defined as the ratio of portfolio risk measure from the portfolio strategies (17) and (18) to that from (7) and (8) respectively. The other portfolio selection strategies in literature’s risk reduction are estimated as the ratio of portfolio variance to that from Markowitz’s mean–variance framework.

Sparsity may result in under-diversification and extreme weights of the portfolio. We therefore present the average number of a subset of assets with non-zero weights. We consider another performance metric known as portfolio turnover. A portfolio turnover measures the frequency with which assets in this case stocks are bought and sold. We employ the measure used by DeMiguel et al. (2009) and Kourtis et al. (2012) by defining the turnover rate of the portfolio between *t* to $t+1$ as

22 $$\begin{aligned} Turnover= \frac{1}{\mathring{T}-T-1}\sum _{t=T}^{\mathring{T}-1}\sum _{k=1}^N(\mid x_{k,t+1}-x^-_{k,t+1}\mid ), \end{aligned}$$

where $x_{k,t+1}$ is the portfolio weight for stock *k* at $t+1$, $x^-_{k,t+1}$ is the portfolio weight before revision at $t+1$, ${\mathring{T}-T-1}$ represents the length of the non-zero elements in total portfolio return and *N*, the number of stocks.

The introduction of transaction costs affects the overall profitability of a portfolio strategy. In practice, they lessen the net returns and diminish capital available for future investments. We assume proportional transaction cost for the purpose of this study (Please refer to Assumption 1 for more details). We investigate the impact of transaction cost on our portfolio strategy by computing Sharpe ratio with transaction cost. In particular, the optimal portfolios can be obtained by solving problems (17) and (18). To highlight the effect of transaction costs, we consider two situations with transaction costs, $c^b_k=c^s_k=0$ and $c^b_k=c^s_k=0.02$.

### Computational results

Tables 2 and 3 shows the annualized out-of-sample metrics for different periods of each portfolio considered in this study: RSMt is the regularized mean-safety first portfolio with transaction costs, RSMVt is the regularized mean–variance-safety-first portfolio with transaction costs. The others are selected alternative portfolio strategies in literature.

**Table 2:** Out-of-sample performance of portfolio strategies on 500 NYSE stocks of sub-sample periods

Portfolio selection strategy	SRatio (transaction costs)	SRatio	Risk reduction	Sparsity	Turnover
*Panel A : results for January 2006 to December 2008*					
RSMt (17)	0.6891	0.6902	0.2115	165.1612	0.1517
RSMVt (18)	0.7111	0.7209	0.1801	188.6575	0.1783
minVu	−0.1523	0.1902	0.2561	500	0.4013
minVc	0.2042	0.2821	0.2393	500	0.4209
MV	−0.1745	0.1887	1.0000	500	4.1910
1/*N*	0.4084	0.4810	0.2710	500	0.3575
$l_1$-MV (Brodie et al. 2009)	0.41014	0.5131	0.2604	217.1435	0.3872
TMN (Tu and Zhou 2011)	−0.0158	0.3412	0.2573	500	0.4643
VD (Kirby and Ostdiek 2012)	0.3914	0.4710	0.2104	500	0.4510
*Panel B : results for January 2009 to December 2011*					
RSMt (17)	1.2319	1.2783	0.0932	151.2341	0.0613
RSMVt (18)	1.3012	1.3114	0.0815	203.2533	0.0791
minVu	0.0985	0.4612	0.1044	500	0.2002
minVc	0.4202	0.5319	0.1012	500	0.2125
MV	0.0812	0.4104	1.0000	500	2.1234
1/*N*	0.7264	0.8041	0.1395	500	0.1453
$l_1$-MV (Brodie et al. 2009)	0.9011	0.9511	0.1391	326.5532	0.1877
TMN (Tu and Zhou 2011)	0.2216	0.6013	0.1091	500	0.2563
VD (Kirby and Ostdiek 2012)	0.8153	0.8921	0.0995	500	0.2418
*Panel C : results for January 2012 to December 2015*					
RSMt (17)	1.3112	1.3205	0.0901	195.0186	0.0717
RSMVt (18)	1.3813	1.3984	0.0737	245.1227	0.0801
minVu	0.1120	0.4824	0.1018	500	0.2011
minVc	0.4413	0.5302	0.1006	500	0.2289
MV	0.1091	0.4591	1.0000	500	2.3914
1/*N*	0.7641	0.8321	0.1391	500	0.1465
$l_1$-MV (Brodie et al. 2009)	0.9403	0.9821	0.1294	267.8271	0.1903
TMN (Tu and Zhou 2011)	0.2426	0.63841	0.1067	500	0.2613
VD (Kirby and Ostdiek 2012)	0.8534	0.9010	0.0945	500	0.2485

Performance measures and metrics of portfolio selection strategies

**Table 3:** Out-of-sample performance of portfolio strategies for 2009–2015

Portfolio selection strategy	SRatio (transaction costs)	SRatio	Risk reduction	Sparsity	Turnover
*Results for January 2009 to December 2015*					
RSMt (17)	0.8912	0.9111	0.1521	175.5103	0.9859
RSMVt (18)	0.9577	0.9610	0.1112	228.3181	0.8113
minVu	0.0698	0.2413	0.1742	500	1.1612
minVc	0.2408	0.2958	0.1663	500	1.6209
MV	0.0451	0.1959	1.0000	500	7.3224
1/*N*	0.5514	0.5911	0.1977	500	0.9418
$l_1$-MV (Brodie et al. 2009)	0.6899	0.7094	0.1790	254.8511	0.9958
TMN (Tu and Zhou 2011)	0.1354	0.2985	0.1551	500	1.4705
VD (Kirby and Ostdiek 2012)	0.4544	0.5871	0.1605	500	1.2403

Performance measures and metrics of portfolio selection strategies

Analyzing portfolio risk, the regularized portfolio rules have lower risk than other portfolio selection strategies. This can be attributed to the downside risk measure, Roy safety-first principle considered in the regularized strategies. The variance and the safety-first principle as a consolidated risk measure in RSMVt have lower risk reduction compared to RSMt.

The portfolio turnover indicates how frequently assets in a portfolio are bought and sold. This performance measure is preferred to be small. In terms of turnover, the naive 1/*N* portfolio has the lowest turnover. The two portfolio selection strategies considered in this paper have relatively low turnover rates with RSMVt the preferred choice. The portfolio rules in selected from literature have a high turnover rate as compared to the regularized models in this paper. The highest turnover comes from TMN, MV and minVu portfolios leading to smaller Sharpe ratios. We observe that with large turnovers, feasible transaction costs lowers the monetary gains of many selected portfolios strategies in literature, as seen by Sharpe ratio deductions after transaction costs have been considered.

The Sharpe ratio allows investors to analyse risk-adjusted returns in exchange for the level of risk they are assuming. The higher the Sharpe ratio, the more returns the investor gets per unit of risk. The lower the Sharpe ratio, the more risk the investor bears to get more returns. Comparing all strategies in this study, the regularized portfolios have the highest Sharpe ratios with RSMVt leading the way. With regards to sparsity, more than 30 % of stocks are selected by RSMt and RSMVt in all the evaluation periods. The $l_1$-penalized mean–variance model selects at least 50 % of the stocks across the evaluation periods. The smaller set of sparse portfolio optimizes the budget allocation by focusing on stocks believed to foster diversification.

From Table 2, the sample-based portfolios i.e. minVu, minVc, MV and TMN perform worse due to a large number of stocks that increases the degree of estimation risk or over-fitting. Apart from VD, all other non-regularized portfolios selected from literature (minVu, minVc, MV, TMN) perform worse than 1/*N* regarding both Sharpe ratio and turnover. We observe an increment in Sharpe ratio, lower risk reduction, lower turnover for post financial crisis sub-sample periods. Among the selected alternative portfolio strategies considered in this paper, $l_1$MV has a better out-of-sample performance than minVu, minVc, MV, 1/*N*, TMN and VD. In comparing periods 2006–2008 to 2009–2015, we observe that during the financial crisis period, the Sharpe ratio is lower and the risk reduction is much higher with turnover ratio also higher. This amounts to lower returns during 2006–2008 as compared to the other periods. The Sharpe ratio is much higher and the risk reduction is lower in periods 2009–2011 and 2012–2015 as compared to periods 2006–2008 and 2009–2015. In all the sub-sample periods 2006–2008, 2009–2011 and 2012–2015 including the period 2009–2015, our proposed models RSMt and RSMVt have better out-of-sample performance than the selected alternative models.

**Fig. 1:**
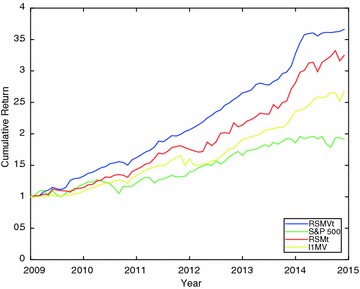
Cumulative wealth of portfolio strategy RSMt, RSMVt and $l_1$MV relative to S&P 500 portfolio

To further gain financial insights, we use S&P 500 index data from January 2009 to December 2015 as a benchmark portfolio. With a starting wealth value of $1, we compare the cumulative wealth of portfolio strategies RSMt, RSMVt and $$l_1$$MV to that of S&P 500 index. To provide evidential proof, Fig. 1 plots the cumulative wealth of the portfolios strategies relative to S&P 500 benchmark.

In Fig. 1, $1 is used as initial wealth and it grows at a monthly return of the portfolio selection strategies considered. The figure shows distinctly the higher performance of the regularized portfolio over S&P 500 benchmark.

## Conclusion

In this paper, we seek near-optimal sparse and stable portfolios to reduce the difficulty of portfolio management. Theoretical results are established to guarantee the stability and sparsity of our novel portfolio strategies. Computational evidence indicates that $l_1$- squared $l_2$ penalized mean-Roy safety-first portfolio and $l_1$- squared $l_2$ penalized mean–variance-Roy safety-first portfolio are able to choose optimal sparse and stable portfolios while maintaining satisfactory out-of-sample performance.

We compare the performance of our proposed models ($l_1$- squared $l_2$ penalized mean-Roy safety-first portfolio and $l_1$- squared $l_2$ penalized mean–variance-Roy safety-first portfolio) of optimal asset allocation relative to selected alternative portfolio strategies in literature (minVu, minVc, MV, 1/*N*, $l_1$MV, TMN and VD). Our results show that our regularized proposed models have a better out-of-sample performance with high Sharpe ratios and relatively low turnover rates. Except for VD, the Sharpe ratio of 1/*N* portfolio when compared to other selected non-regularized portfolio rules considered in this paper is higher, which shows that estimation errors in returns shrink gains from other selected classical portfolio strategies in literature. The norm penalty improves Sharpe ratio and turnover. As a result, $l_1$MV has a better out-of-sample performance than minVu, minVc, MV, 1/*N*, TMN and VD.

To gain more financial acumen, we compare our proposed models and the best performing portfolio strategy among the selected models from literature considered in this paper, to a benchmark, S&P 500 index. The results indicate that given an initial wealth of $1, the excess returns from $$l_1$$- squared $$l_2$$ penalized mean–variance-Roy safety-first portfolio is the highest. Our proposed models for optimal asset allocation are favourable since they overcome unsteady and extreme portfolio weights induced by estimation error due to parameter uncertainty.

## References

[CR1] Bawa VS, Brown SJ, Klein RW (1979) Estimation risk and optimal portfolio choice. North-Holland Publ Co, New York

[CR2] Bawa VS (1978). Safety-first, stochastic dominance, and optimal portfolio choice. J Financ Quant Anal.

[CR3] Borkovec M, Domowitz I, Kiernan B, Serbin V (2010). Portfolio optimization and the cost of trading. J Invest.

[CR4] Brandt M (2009). Portfolio choice problems. Handbook Financ Econom.

[CR5] Britten-Jones M (1999). The sampling error in estimates of mean-variance efficient portfolio weights. J Finance.

[CR6] Brodie J, Daubechies I, De Mol C, Giannone D, Loris I (2009). Sparse and stable Markowitz portfolios. Proc Natl Acad Sci.

[CR7] Chang TJ, Meade N, Beasley JE, Sharaiha YM (2000). Heuristics for cardinality constrained portfolio optimisation. Comput Oper Res.

[CR8] Chiu MC, Wong HY, Li D (2012). Roys safety-first portfolio principle in financial risk management of disastrous events. Risk Anal.

[CR9] DeMiguel V, Garlappi L, Nogales FJ, Uppal R (2009). A generalized approach to portfolio optimization: improving performance by constraining portfolio norms. Manag Sci.

[CR10] DeMiguel V, Mei X, Nogales FJ (2014) Multiperiod portfolio optimization with many risky assets and general transaction costs. Available at SSRN 2295345

[CR11] Dumas B, Luciano E (1991). An exact solution to a dynamic portfolio choice problem under transactions costs. J Finance.

[CR12] Dybvig PH (2005) Mean-variance portfolio rebalancing with transaction costs. Working paper, Washington University in Saint Louis

[CR13] Fan J, Zhang J, Yu K (2012). Vast portfolio selection with gross-exposure constraints. J Am Stat Assoc.

[CR14] Farrell JL, Reinhart WJ (1997). Portfolio management: theory and application.

[CR15] Goldfarb D, Iyengar G (2003). Robust portfolio selection problems. Math Oper Res.

[CR16] Green RC, Hollifield B (1992). When will mean-variance efficient portfolios be well diversified?. J Finance.

[CR17] Haque M, Varela O, Hassan MK (2007). Safety-first and extreme value bilateral US–Mexican portfolio optimization around the peso crisis and NAFTA in 1994. Q Rev Econ Finance.

[CR18] Jagannathan R, Ma T (2003). Risk reduction in large portfolios: why imposing the wrong constraints helps. J Finance.

[CR19] Jorion P (1986). Bayes–Stein estimation for portfolio analysis. J Financ Quant Anal.

[CR20] Kellerer H, Mansini R, Speranza MG (2000). Selecting portfolios with fixed costs and minimum transaction lots. Ann Oper Res.

[CR21] Kirby C, Ostdiek B (2012). It’s all in the timing: simple active portfolio strategies that outperform naive diversification. J Financ Quant Anal.

[CR22] Kourtis A, Dotsis G, Markellos RN (2012). Parameter uncertainty in portfolio selection: shrinking the inverse covariance matrix. J Bank Finance.

[CR23] Ledoit O, Wolf M (2004). A well-conditioned estimator for large-dimensional covariance matrices. J Multivar Anal.

[CR24] Levy H, Sarnat M (1972). Safety firstan expected utility principle. J Financ Quant Anal.

[CR25] Lobo MS, Fazel M, Boyd S (2007). Portfolio optimization with linear and fixed transaction costs. Ann Oper Res.

[CR26] Maringer D, Kellerer H (2003). Optimization of cardinality constrained portfolios with a hybrid local search algorithm. OR Spectrum.

[CR27] Markowitz H (1952). Portfolio selection. J Finance.

[CR28] Markowitz HM (1968). Portfolio selection: efficient diversification of investments.

[CR29] Merton RC (1980). On estimating the expected return on the market: an exploratory investigation. J Financ Econ.

[CR30] Mitchell JE, Braun S (2013) Rebalancing an investment portfolio in the presence of convex transaction costs, including market impact costs. Optim Methods Softw 28(3):523–542

[CR31] Moral-Escudero R, Ruiz-Torrubiano R, Suárez A (2006) Selection of optimal investment portfolios with cardinality constraints. In: IEEE congress on evolutionary computation, CEC 2006, IEEE; pp 2382–2388

[CR32] Muthuraman K, Kumar S (2006). Multidimensional portfolio optimization with proportional transaction costs. Math Finance.

[CR33] Nagai H (2003). Optimal strategies for risk-sensitive portfolio optimization problems for general factor models. SIAM J Control Optim.

[CR34] Nawrocki DN (1999). A brief history of downside risk measures. J Invest.

[CR35] Pai G, Michel T (2009). Evolutionary optimization of constrained-means clustered assets for diversification in small portfolios. IEEE Trans Evol Comput.

[CR36] Platen E, Heath D (2006). A benchmark approach to quantitative finance.

[CR37] Rachev ST (2001). Safety-first analysis and stable paretian approach to portfolio choice theory. Math Comput Model.

[CR38] Rachev S, Ortobelli S, Stoyanov S, Fabozzi FJ, Biglova A (2008). Desirable properties of an ideal risk measure in portfolio theory. Int J Theor Appl Finance.

[CR39] Rockafellar RT, Uryasev S (2000). Optimization of conditional value-at-risk. J Risk.

[CR40] Roman D, Darby-Dowman K, Mitra G (2007). Mean-risk models using two risk measures: a multi-objective approach. Quant Finance.

[CR41] Roy AD (1952) Safety first and the holding of assets. Econom J Econom Soc 20:431–449

[CR42] Ruiz-Torrubiano R, Suárez A (2015). A memetic algorithm for cardinality-constrained portfolio optimization with transaction costs. Appl Soft Comput.

[CR43] Schultz R, Tiedemann S (2003). Risk aversion via excess probabilities in stochastic programs with mixed-integer recourse. SIAM J Optim.

[CR44] Sharpe WF (1966). Mutual fund performance. J Bus.

[CR45] Telser LG (1955). Safety first and hedging. Rev Econ Stud.

[CR46] Tibshirani R (1996) Regression shrinkage and selection via the lasso. J R Stat Soc Ser B (Methodol) 1:267–288

[CR47] Tütüncü RH, Koenig M (2004). Robust asset allocation. Ann Oper Res.

[CR48] Tu J, Zhou G (2011). Markowitz meets Talmud: a combination of sophisticated and naive diversification strategies. J Financ Econ.

[CR49] Zou H, Hastie T (2005). Regularization and variable selection via the elastic net. J R Stat Soc Ser B (Stat Methodol).

